# Incorporating Novel Mobile Health Technologies Into Management of Knee Osteoarthritis in Patients Treated With Intra-Articular Hyaluronic Acid: Rationale and Protocol of a Randomized Controlled Trial

**DOI:** 10.2196/resprot.5940

**Published:** 2016-08-09

**Authors:** Donald Jones, Nebojsa Skrepnik, Richard M Toselli, Bruno Leroy

**Affiliations:** ^1^ Scripps Translational Science Institute La Jolla, CA United States; ^2^ Tucson Orthopedic Institute Research Center Tucson, AZ United States; ^3^ Sanofi-Aventis Paris France

**Keywords:** mHealth, osteoarthritis, pain, physical therapy

## Abstract

**Background:**

Osteoarthritis (OA) of the knee is one of the leading causes of disability in the United States. One relatively new strategy that could be helpful in the management of OA is the use of mHealth technologies, as they can be used to increase physical activity and promote exercise, which are key components of knee OA management.

**Objective:**

Currently, no published data on the use of a mHealth approach to comprehensively monitor physical activity in patients with OA are available, and similarly, no data on whether mHealth technologies can impact outcomes are available. Our objective is to evaluate the effectiveness of mHealth technology as part of a tailored, comprehensive management strategy for patients with knee OA.

**Methods:**

The study will assess the impact of a smartphone app that integrates data from a wearable activity monitor (thereby both encouraging changes in mobility as well as tracking them) combined with education about the benefits of walking on patient mobility. The results from the intervention group will be compared with data from a control group of individuals who are given the same Arthritis Foundation literature regarding the benefits of walking and wearable activity monitors but who do not have access to the data from those monitors. Activity monitors will capture step count estimates and will compare those with patients’ step goals, calories burned, and distance walked. Patients using the novel smartphone app will be able to enter information on their daily pain, mood, and sleep quality. The relationships among activity and pain, activity and mood, and sleep will be assessed, as will patient satisfaction with and adherence to the mobile app.

**Results:**

We present information on an upcoming trial that will prospectively assess the ability of a mobile app to improve mobility for knee OA patients who are treated with intra-articular hyaluronic acid.

**Conclusions:**

We anticipate the results of this study will support the concept that mHealth technologies provide continuous, real-time feedback to patients with OA on their overall level of activity for a more proactive, personalized approach to treatment that may help modify behavior and assist with self-management through treatment support in the form of motivational messages and reminders.

## Introduction

### Osteoarthritis Management and Mobility

Osteoarthritis (OA), a prevalent condition afflicting nearly 27 million US adults, is one of the leading causes of disability in the United States [1,2]. Pain and the other symptoms associated with OA of the knee have a profound negative impact on patients’ quality of life, affecting both physical activity and psychological well-being [3-5]. Limitations in walking, stairclimbing, and kneeling are common and greatly interfere with activities of daily living and recreation, which may have negative effects on patients’ social interactions, sleep, and mental functioning [4].

The primary goals of most treatments for OA are to reduce pain and improve the function of the affected joints [6,7]. Optimal management of OA requires a combination of both pharmacologic and nonpharmacologic methods to achieve these goals [6-8]. Pharmacologic methods include oral or topical analgesics or intra-articular therapies, such as viscosupplementation, that focus on reducing pain, whereas nonpharmacologic methods include weight loss, physical activity, and exercise [6-9]. Although, there is no standard program of education or exercise and no clear benefit of one exercise program compared with another [7], 1 study found that a regular walking program can be associated with reduced pain and improved quality of life [10], while another study has demonstrated that walking can significantly reduce the risk of functional limitations in patients with knee OA [11]. This suggests that walking, which is an intrinsic activity of daily living, by itself can have a substantial impact on reducing symptoms of OA and maintaining function. Multiple societal guidelines also recognize patient education and self-management strategies as important components of knee OA management [6-8]. When implemented in conjunction with weight loss and exercise activity, these programs can increase adherence to prescribed management paradigms and may increase the overall effectiveness of treatment [12].

Unfortunately, the majority of patients do not routinely incorporate exercise or physical activity into their lives, despite their well-known benefits [13]. Patients with knee OA have been found to be particularly sedentary and being less sedentary has been associated with better physical function in these patients [14]. Increasing mobility and decreasing sedentary behavior may also lead to improved blood pressure, reduced cardiovascular risk [15], and improved quality of life [16]. One relatively new strategy that may be used to enhance OA management and increase physical activity is the use of mHealth technologies (ie, the use of mobile apps for health care). These technologies can provide continuous feedback to patients on their overall level of activity; they can also assist with self-management by providing treatment support in the form of motivational messages and reminders. To date, however, no published data are available on the use of mHealth technologies in the management of knee OA. Research is thus needed on the potential effectiveness of mHealth technologies as part of a tailored, comprehensive management strategy.

### mHealth Technologies and Limitations: Brief Background

This brief section is intended for the clinical audience who may be less familiar with mHealth technologies. The development of mHealth devices, apps, social media, and Web-based services is leading to a paradigm shift in health care, and these technologies can serve a multitude of functions, such as facilitating customized communication to or between health care providers and consumers, collecting data, and delivering care. They can also provide individual level support for both health care providers (eg, education) and consumers (eg, appointment or medication reminders or test result notifications) [17]. Wearable devices, such as bracelets, watches [18,19], skin patches/strips [20,21], and even “smart” apparel, which contain biosensors [22-24], can now be used to monitor, track, and transmit health metrics continuously and in real time. Personalized information can be provided to guide health and wellness that may facilitate interactive, individualized treatment and give feedback to motivate patients to adopt a new behavior or excel in an existing activity. Increasingly, smartphones themselves are providing feedback by using the embedded sensors that have become standard fare to capture data. It is not unusual to combine these data sources with information from external sources, such as Google Maps, Google Traffic, National Oceanic and Atmospheric Administration, and even Facebook to deliver actionable information to the patient. mHealth also empowers patients with user-friendly information, which allows them to have more say and take more responsibility for their treatment.

mHealth has the potential to provide numerous benefits for patients, physicians, and other key individuals involved in health care delivery [25]. One of the most common apps is a reminder for patients to take their medications (via either phone calls or SMS text messaging (short message service, SMS)), but mobile phones can also be used to deliver insurance information, as well as increase the efficiency of emergency services and responses. The Web-based pharmacy, Walgreens, offers one of the most successful mHealth mobile apps, which provides reminders to take medications, facilitates requests for medication refills, and is now used by millions in the United States while providing Walgreens loyalty points for doing so. mHealth services can also promote healthy behavior and provide real-time patient monitoring and communication. All of the above could improve the overall efficiency of health care by reducing costs.

Unfortunately, the overall quality of the data gathered by these technologies is low. A systematic review and meta-analysis of controlled trials of the effectiveness of mobile technology interventions conducted in 2013 concluded that none were high-quality trials [17]. The majority of the trials examined in this review tested interventions directed at health care providers, not patients, with mixed results, and none reported any objective clinical outcome. Many of the studies focused on the impact of using SMS text messaging either to provide information, such as test notifications and reminders for appointments, or to affect patient behavior, such as treatment adherence or smoking cessation [26-31], yet failed to provide sufficient evidence of the benefit of these apps.

The nature of the data captured by these devices is different from classical clinical data, as it is patient-generated and not directly observed by clinical researchers. Currently, no standards or guidelines have been developed to interpret data from wearables or how to transform it into data that are quantifiable and clinically meaningful. The data tend to be available as streams rather than traditional clinical episodic data points, and researchers are unsure about how to relate trending data to other data points. Data from these types of devices and apps also need to be put into the proper context. For example, a device may indicate that a patient’s mobility is increased, but increased mobility does not necessarily mean that a patient is feeling better and may not even be the best indicator of improvement.

Devices have varying capabilities, do not measure activities in the same way, apps are often device-specific, and the need for software/hardware updates is common, making comparison a technical challenge. Different devices have various combinations of accelerometers, gyroscopes, and electronic compasses. Some have built-in global positioning system technology or access location data from paired smartphones. Sampling rates, processor power, and motion data analytic software capabilities vary widely. Many are associated with false-positive and false-negative results. Activities such as horseback riding can register as substantial activity or movement, whereas riding a stationary bike may register only as minimal activity (or none at all). Most current devices are unable to capture water-based activities. To capture data correctly, patients must be compliant and wear or use the device as directed, but it is impossible to confirm that the individual is actually wearing or using the device.

Finally, there are financial barriers with regard to implementation of mHealth technologies. Although 91% of the adults in the United States own a mobile phone, with 61% of these individuals owning a smartphone [32], a portion of the population still cannot afford such a device. The current fee-for-service health care model also hinders uptake of mHealth technologies, with little financial incentive for practitioners to implement preventive care; and value-based care is not foreseen to overtake fee-for-service care until 2020. An exception to this paradigm is the upstart insurer Oscar Health, which received a $32.5 million investment from Google in 2015 and provides its insured customers with activity monitors and financial incentives to use them. Oscar Health operates in New York and New Jersey and will expand into California and Texas in 2016. Many self-insured employers are also making activity monitoring and feedback a central component of their corporate wellness programs.

### Overview of mHealth in Support of Osteoarthritis Management

Innovative mHealth technologies specifically designed for patients with chronic conditions such as OA may be particularly useful. They can be used to help change behavior or improve (and possibly simplify) disease management (eg, by increasing adherence to prescribed medication) and can provide definitive objective information (eg, “I walked 5000 steps yesterday” or “I walked 15% more this week over last week”) rather than subjective feedback (eg, “I was as active as I normally am”), which would be beneficial, as many of the tools used for assessments in OA, such as visual analog scales, are subjective. Patients from different backgrounds (geographic locations as well as ethnic backgrounds) have different, subjective perceptions of pain. Developing objective assessment measures for OA would thus be an advance in terms of assessing patients’ physical status as well as the effectiveness of new treatments. Furthermore, the symptoms of OA are often variable depending on patients’ activity level, changes in weather, and various other factors. Patients may not have pain at rest but can have severe pain during activities such as climbing stairs. As such, validated, documented questionnaires, such as the Western Ontario and McMaster Universities Osteoarthritis Index (WOMAC 3.1), which is used to assess pain, stiffness, and physical function over a specific time frame (ie, the last 48 hours) in clinical trials [33], can completely miss the effect of treatment. For instance, a patient in a clinical trial of treatment for knee OA may present for a regularly scheduled study visit at week 12, noting that pain over the last 4 weeks has been low, whereas pain over the last 3 days (when the patient has been gardening and shopping and/or there has been a dramatic weather change) has been high. The opposite scenario could also occur: a patient may feel unwell for several weeks and then decide to rest, so the target knee on the day of assessment may feel good. mHealth technologies may allow establishment of baseline activity or pain levels (eg, in patients where movement is impaired by pain) so that any change in real time over the course of the study may be more objectively assessed and treatment adjustments can be made. Finally, reported pain can be indexed to actual activity to see the personalized correlation between reported pain and activity.

mHealth technologies have the potential to provide more continuous, real-time monitoring of patient health, which would be helpful for patients with chronic conditions, such as OA, as it may provide a more proactive, personalized approach to treatment. For example, patients with diabetes would greatly benefit from continuous monitoring of blood glucose to facilitate changes in treatment, as would cardiac patients with arrhythmias if changes in heart rate could be captured with a mobile device. In fact, the Dexcom Mobile Continuous Glucose Monitoring system [34] includes an activity sensor that reports activity to the patient as part of its therapy management system, and the iRhythm ZIO XT [21] patch can provide continuous monitoring of cardiac arrhythmias [35] and is preferred by patients over traditional Holter monitors [36]. Patients with OA could similarly benefit from continuous monitoring of activity and pain. There are already beds and pillows with sensors that capture body movement and position during sleep for assessment of sleep apnea, floors with sensors that capture changes in gait (for fall prevention), and digital cameras and programs that analyze skin color for assessment of liver, kidney, or cardiac diseases. Although many of these devices are prototypes, they will likely lead to improved, more reliable, and more sophisticated technologies. Patients may be particularly open to the use of mHealth technologies in the management of their OA for many of the reasons above. In one recent study of attitudes toward wearable technology, patients with knee OA expressed the perception that wearable technology could positively benefit their sense of control over their condition, improve awareness of progress and communication with their clinician, and empower their self-management [37].

### Development of Evidence to Assess Effectiveness of mHealth

Evidence is accumulating regarding the effectiveness of mHealth interventions. Two systematic reviews have described a robust evidence base for the use of SMS text message reminders to improve attendance at health care appointments [26,38]. Another systematic review found that mobile app–based interventions promote weight loss [39]. Increased physical activity following use of Internet- and mobile app–based interventions has been observed in several studies such as those promoting a healthy lifestyle [40], workplace-based increased walking [41], engagement in regular physical activity in cancer survivors [42], maintenance of physical activity after cardiac rehabilitation [43], and post-rehabilitation exercise persistence in patients with chronic obstructive pulmonary disease [44]. Conversely, however, a systematic review of studies that used the Internet to deliver the primary component of treatment has shown only modest effects on health-related behavior [45], and a recent systematic review of the effectiveness of online social network health behavior interventions found modest benefits at best (most were small in magnitude and nonsignificant) [46].

At this time, no published data are available on the use of a mHealth approach to comprehensively monitor physical activity in patients with OA, and no data have been published on whether mHealth technologies can impact outcomes. Studies have shown that regular phone contact can improve the clinical status of patients with knee OA [6,47]. For example, a randomized controlled trial in 439 patients with OA demonstrated that monthly phone contact aimed at promoting self-care for patients with knee OA could be associated with improvements in joint pain and physical function [47]. This suggests that smartphone apps may be helpful in improving outcomes in patients with knee OA. A new clinical trial (presented herein) will prospectively investigate this hypothesis by assessing the ability of a mobile app to improve the mobility of patients with knee OA who are treated with hylan G-F 20, an elastoviscous high molecular weight fluid containing hylan A and B polymers that is approved for the treatment of pain in moderate to severe OA of the knee [48].

### MARCHE Study Rationale

**Figure 1:**
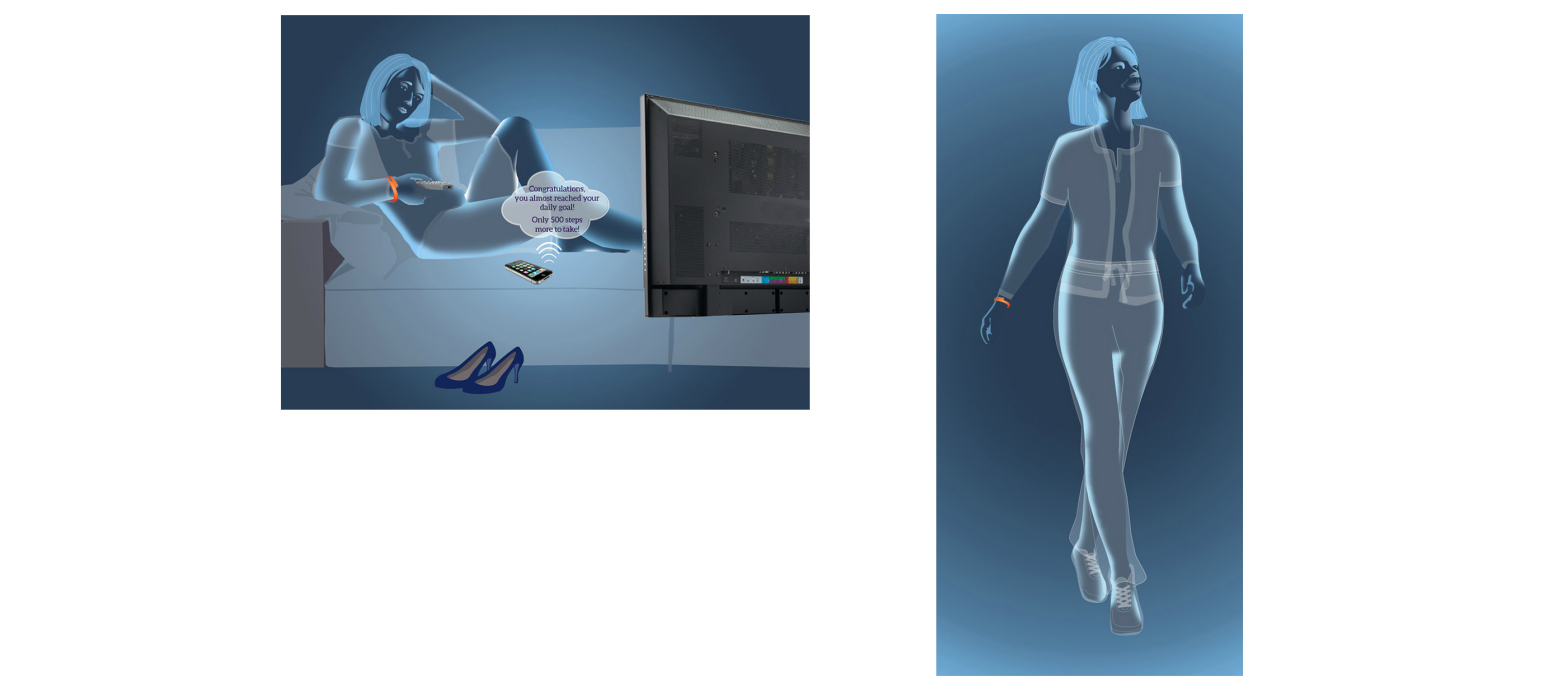
Conceptualization of the MARCHE clinical trial. Study participant (a) receives a motivational message from the OA GO smartphone application and then (b) begins to walk.

The MARCHE trial was conceptualized to help patients with knee OA connect management of their mobility and monitoring of their functional abilities with self-management strategies. The study is designed to assess the impact of a smartphone app (which encourages changes in mobility) that integrates data from a wearable activity monitor (which will capture changes in mobility) with education about the benefits of walking (Figure 1). The data will be compared with those from a control group of individuals who are given Arthritis Foundation literature regarding the benefits of walking and who do not have access to the data from their wearable activity monitors. The activity monitors will capture estimates of step counts, comparing them with patients’ step goals, calories burned, and distance walked, whereas the novel smartphone app will allow patients to enter information on their daily pain, mood, and sleep quality. Assessment of the relationships among activity and pain, activity and mood, and sleep will be performed, as will assessment of patient satisfaction with and adherence to the mobile app. Data will be indexed to time of day and date.

The activity monitor and mobile app will allow continuous monitoring of patient activity levels and will be a more objective parameter compared with established and validated scores, such as WOMAC and visual analog scales. Patients with OA may indicate that they would like to be more active and are aware of the benefits of exercise, but that they are limited by pain. Being able to actively, objectively, and continuously monitor activity levels could provide key insights in personalizing patients’ treatment regimens.

**Patient inclusion criteria.**

• Must have osteoarthritis of the knee, which the investigator decided to treat with hylan G-F 20 according to the approved label in the United States.

• Must be able to read, understand, and sign an informed consent form, understand requirements for follow-up visits, and provide information at the    scheduled evaluation.

• Must be able to read and understand English.

**Patient exclusion criteria.**

• Age <30 or >80 years.

• Being pregnant or currently breast-feeding (women of childbearing potential not protected by highly effective contraception also excluded).

• Unfamiliarity with smartphones.

• Baseline pain in target-for-treatment knee while walking on a flat surface >9 on 11-point numeric rating scale.

• Bilateral disease (but may be included if only one knee is treated and contralateral knee pain must be <4 while walking on a flat surface on 11-point    numeric rating scale).

• Body mass index >35.

• Short life expectancy (<12 months).

• Current use of Jawbone, Fitbit, or any other wearable activity monitor or an analogous device.

• Ongoing litigation or workers’ compensation claim related to knee pain.

• Surgery on any lower extremity joint.

• Significant medical condition or other factor that investigator would feel would interfere with study evaluation or participation.

• Chronic narcotic use.

• Daily step average <500 or >8000.

## Methods

### Patient Eligibility Criteria and Study Design

Patient inclusion and exclusion criteria are listed in Textboxe 1 and Textboxe 2. The inclusion criteria were designed to identify patients who were candidates for treatment with hylan G-F 20 and would be capable of using the wearable activity monitor and a smartphone.

The MARCHE study is a randomized (1:1), open-label, multicenter, parallel-group study in patients with OA of the knee treated with hylan G-F 20. The trial will be conducted in accordance with the Declaration of Helsinki and the International Conference on Harmonisation Guideline for Good Clinical Practice, and each investigator will obtain institutional review board or ethics committee approval. The primary objective of the study is to demonstrate the impact of a mobile app (OA GO) plus a wearable activity monitor in increasing the mobility of patients with knee OA who were treated with hylan G-F 20. Secondary objectives include evaluating the impact of the OA GO app combined with the wearable activity monitor on a 6-minute walk test, assessing patient and physician satisfaction with the OA GO app and the wearable activity monitor, determining the percent change from baseline in steps per day, and determining between-group changes from baseline in the Patient Activation Measure-13 (PAM-13), which assesses patient activation regarding health self-management. Relationships among mobility, pain, mood, and sleep will also be established.

**Figure 2:**
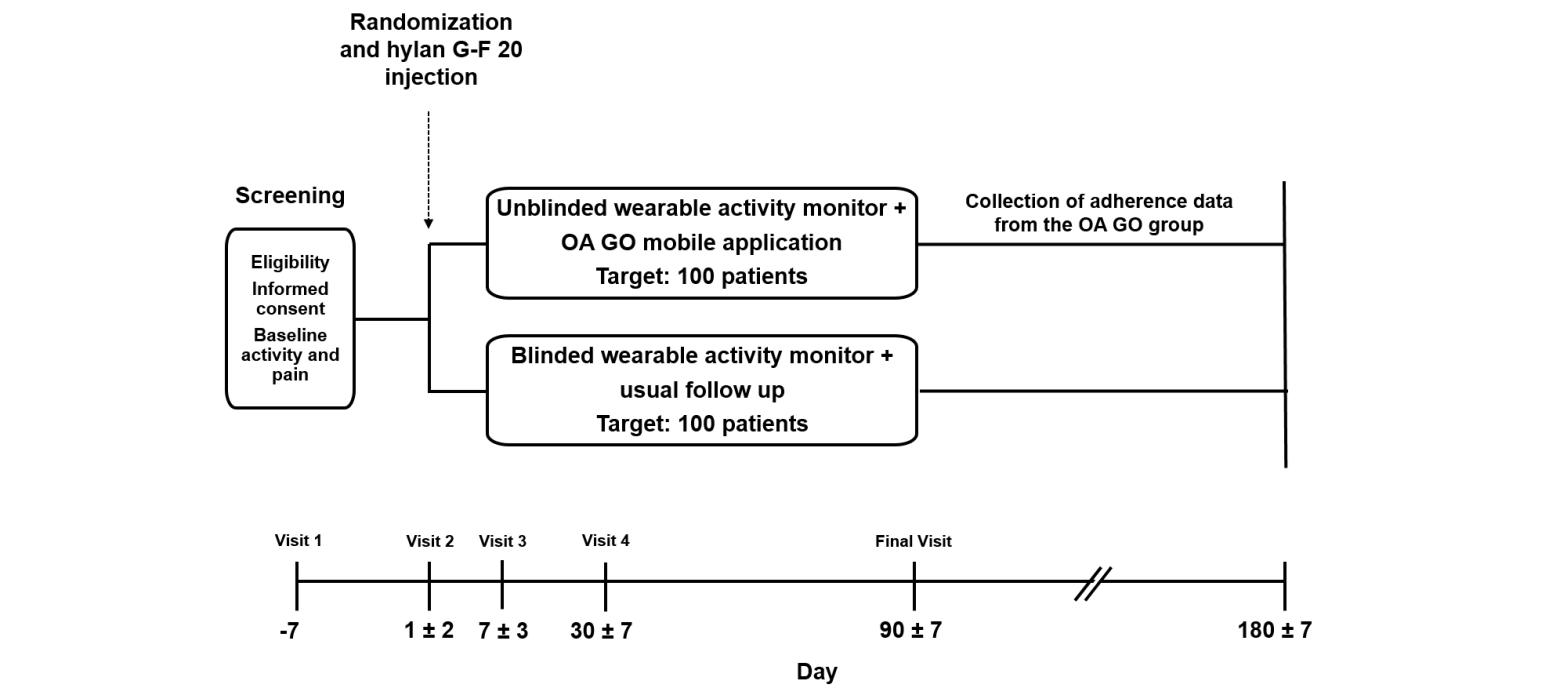
Design of the MARCHE clinical trial.

Figure 2 shows the study protocol. Patients who are selected by the investigator for treatment with hylan G-F 20 for OA of the knee will be screened for eligibility, and their baseline pain will be assessed. Patients will also be required to provide written informed consent. If eligible, patients will be given a wearable activity monitor (Jawbone UP24) that will record their daily steps and other variables over the next 7 days (to establish their baseline activity level). Jawbone UP24 is a commercially available wearable activity monitor designed to be worn on the wrist. After at least 7 days, patients will return for assessment of baseline activity, mood (using Visual Analog Mood Scales (VAMS) [49]), and a baseline 6-minute walk test. Eligible patients will receive a single intra-articular injection of hylan G-F 20 in the knee and will be randomized to receive either the mobile OA GO app (unblinded and able to view data from their wearable activity monitor on an ongoing basis) or standard-of-care instructions (blinded with no access to activity recorded by the wearable monitor); randomization will be stratified by site. Additional study visits are performed at days 7 and 30, with the final visit at day 90, when the last evaluation of the primary endpoint will be performed, along with assessments of pain, 6-minute walk test, mood (VAMS), PAM-13, and patient/physician satisfaction. At the end of the trial, all data from the mobile OA GO app will be downloaded and patient adherence will be checked.

### Study Treatment Groups

The study consists of 2 treatment groups, both of which will include the wearable activity monitor. Participants in group A will receive the OA GO mobile app, and their wearable activity monitors will be unblinded. They will be able to view the data from their monitors on an ongoing basis. The OA GO mobile app, which will be downloaded to an iPhone (provided by the study sponsor), contains motivational messages and requests that the patients enter their pain and mood on a daily basis. Training on the use of the mobile app will be provided by the study site. The OA GO app will obtain information from the wearable activity monitor and combine the data with data entered by the patient (such as daily pain and mood). In group B (the control arm of the study), the wearable activity monitors will be blinded; that is, patients in group B (who are blinded to their wearable activity monitor) will not have any access to data recorded by the wearable activity monitor. Data will be downloaded by the study team at last visit. Patients in both treatment groups will receive regular follow-up per the standard-of-care information regarding the benefits of walking from the Arthritis Foundation [13].

## Results

### Study Outcomes

The primary study endpoint is the difference in the change in mobility (assessed by steps per day) between groups at the end of 90 days. Baseline steps per day will be the average of the steps recorded on the wearable activity monitor over 7 days during screening, and the final-visit steps per day will be the average of the steps recorded on the wearable activity monitor over the last 7 days for which steps are recorded. Secondary efficacy endpoints include physical function, assessed by the change from baseline in the 6-minute walk test (distance and pain (pain assessed on an 11-point numeric rating scale, ranging from 0-10)), and percent change from baseline in steps per day. The 6-minute walk test is a validated measure sensitive to assessing changes in the physical performance of older adults [50,51], and has been found to be a reliable measure of functional performance in patients following total hip and total knee arthroplasty [52,53].

Patient-reported outcomes include patient and physician satisfaction with treatment (measured by 6 items for each, on a 7-point Likert scale, and a final item assessing likelihood of using the device in the future (patient) and recommending the device (physician)), PAM-13 scores (responses on a Guttman-like scale ranging from 1-4 (strongly disagree to strongly agree), resulting in possible scores from 13-52 and converted to activation scores ranging from 0-100, the highest degree of activation), and patient-entered values for daily pain and mood for those in the mobile OA GO group. The PAM-13 [54,55] is a reliable and valid measure of patient activation, with higher scores associated with increased self-management behaviors, increased self-efficacy, and activation making it useful for evaluating interventions in a clinical setting.

Tertiary endpoints include target knee pain with walking and at rest, as measured by the 11-point numeric rating scale at final visit; mood at final visit (as measured by the VAMS (capturing 8 domains of mood with raw scores transformed into T-scores with a mean of 50 and standard deviation of 10)); pain and mood as entered into the OA GO app by patients in the mobile OA GO group; sleep quality as measured by the wearable activity monitor; and change from baseline in weight. The VAMS is a brief measure of internal mood state validated in both normal and neurologically impaired individuals [56,57]. Analysis of the relationships between activity and pain, activity and mood, and sleep will also be performed. Lastly, adherence to the use of the mobile OA GO app plus the wearable activity monitor over 90 days after completion of the trial (in patients who received the mobile app) will be determined. Adverse events, including device-related complaints, will also be assessed.

### Data Analysis

The target sample size in this study is 200 participants, which assumes an attrition rate of 15%. Based on a two-sided significance level of 5%, the study will have 80% power to detect an average increase of 25% in the change from baseline in steps per day for those in the unblinded wearable activity monitor group using the OA GO mobile app, compared with those in the blinded wearable activity monitor group who did not have the mobile app.

Efficacy outcomes will be analyzed in the modified intent-to-treat population (all randomized patients with baseline and on-treatment values for the primary endpoint from day 30 onward). Analysis of covariance with baseline mean steps per day as covariate and treatment and pooled site as class variables will be used to compare between-group outcomes. Satisfaction surveys at last visit will be summarized. Safety outcomes will be analyzed for all patients provided a wearable activity monitor.

## Discussion

### Summary and Limitations

Adoption of mHealth technologies into the management of knee OA and other chronic diseases that rely on self-management has the potential to improve patient outcomes; however, data to support this potential are needed. The approach of using these new technologies will also need to be validated. As a first step, the MARCHE study evaluates the impact of a mobile app, combined with a wearable activity monitor, on mobility in patients with knee OA treated with hylan G-F 20. The first patient was enrolled in August 2014, and study enrollment has since been completed.

One limitation of the study is that the investigators are not blinded to the intervention, which could bias the delivery of other aspects of care to the patient. In addition, the step count is only an estimate of the patients’ mobility and is specific to the Jawbone device, so it may not be representative of all devices. The study was focused on activity monitoring and could have included an evaluation of WOMAC scores to assess patient subjective evaluation of changes in condition and function. Finally, the 6-minute walk test could be considered less representative of real-life mobility compared with 6 minutes of walking taken at different time intervals. Future studies could use “continuous walk tests” over a 24-hour period, an example of which would include both a 6-minute walk test and a 25-foot walk test that could be continuously run with algorithms in place to discard, for example, periods of rest, sleep, and driving.

The field of mHealth will continue to evolve. In particular, wearable devices may eventually become implantable devices, which are increasingly being used for health purposes.In 2004, the US Food and Drug Administration approved radio frequency identification tags for human implants, although some safety concerns are associated with these tags. They are small, which means they can move under the skin and become difficult to remove. Internally powered tags could also cause electromagnetic interference with medical devices such as defibrillators. Implantable sensors are currently in development for monitoring glucose and free-floating proteins as biomarkers.

### Conclusions

Ultimately, as technology continues to advance, medicine will do so in kind. The MARCHE study may provide the first real-world evidence of the benefits of health technology in the treatment of knee OA and may provide patients with greater control in the management of their disease.

## Abbreviations

OA: osteoarthritis
PAM-13: patient activation measure-13
SMS: short message service
VAMS: visual analog mood scales
WOMAC: Western Ontario and McMaster Universities Osteoarthritis Index
